# Delayed cerebral ischemia after aneurysmal subarachnoid hemorrhage: a narrative review

**DOI:** 10.1055/s-0045-1809885

**Published:** 2025-06-25

**Authors:** Ingrid Pereira Marques, Carolina Rouanet Cavalcanti de Albuquerque, Natalia Vasconcellos de Oliveira Souza, João Brainer Clares de Andrade, Gisele Sampaio Silva, Pedro Kurtz

**Affiliations:** 1 Universidade Federal de São Paulo, São Paulo SP, Brazil.; 2 Instituto D'Or de Pesquisa e Ensino, Rio de Janeiro RJ, Brazil.; 3 Universidade Federal do Rio de Janeiro, Rio de Janeiro RJ, Brazil.; 4 Hospital Israelita Albert Einstein, São Paulo SP, Brazil.; 5 Centro Universitário São Camilo, São Paulo SP, Brazil.; 6 Instituto Tecnológico de Aeronáutica (ITA), São José dos Campos SP, Brazil.; 7 Instituto Estadual do Cérebro Paulo Niemeyer, Rio de Janeiro RJ, Brazil.

**Keywords:** Subarachnoid Hemorrhage, Brain Ischemia, Critical Care, Cerebral Hemorrhage, Intracranial Aneurysm

## Abstract

Aneurismal subarachnoid hemorrhage (aSAH) is a condition with elevated mortality and morbidity, which usually affects a working-age population, leading to a high socioeconomic burden. Among those who survive the initial bleeding, approximately 30% will experience delayed cerebral ischemia (DCI), which is a significant factor in poor outcomes. However, it is potentially reversible if appropriate treatment is promptly initiated. The amount of blood present on the initial computed tomography (CT) scan, assessed through the modified Fisher scale (mFisher), and the patient's neurological status upon admission, are the strongest predictors of DCI. Early prevention is essential and typically involves administration of enteral nimodipine and the maintenance of euvolemia, while other treatment options have limited supporting evidence. Diagnosing remains a challenge, primarily due to its reliance on clinical examinations. This is more pronounced in high-grade aSAH patients who are unconscious or sedated. In such cases, additional methods may be necessary, such as transcranial Doppler (TCD), continuous electroencephalography (cEEG), or CT with perfusion (CTP). Treatment aims to prevent cerebral infarction and poor clinical outcomes, and it is based on hemodynamic optimization, hypertension induction, cardiac output augmentation, and endovascular therapy. Nevertheless, randomized data on DCI management remains scarce, highlighting the urgent need for more studies and a better understanding of this SAH complication. Addressing this gap may lead to more effective preventive strategies and treatments, which is crucial for improving the prognosis of these patients.

## INTRODUCTION

Aneurysmal subarachnoid hemorrhage (aSAH) accounts for 5 to 10% of all strokes, and it is a condition with high morbimortality rates, predominantly affecting a younger population, which leads to significant economic and social impact. 1 Long-term disability affects between a third and half of the survivors globally. 2 3

Delayed cerebral ischemia (DCI) is a complication that occurs in approximately 30% of aSAH patients, most commonly between 3 to 14 days after symptom onset, 4 5 and it represents a leading cause of poor outcomes and increased costs for those who survive the initial bleeding. 6 7 This is a potentially reversible condition that may progress to permanent infarction if not properly treated. Death and permanent neurological deficits occur in 30 to 40% of DCI patients, and these permanent neurological deficits have been an independent predictor of mortality at 6 and 12 months. 8

We herein aim to provide a synthesis and critical review of DCI's pathophysiology, clinical presentation, and management strategies, highlighting which aspects have more impact on patient outcomes. This includes a comprehensive examination of early diagnostic methods, predictors, and the effectiveness of various therapeutic interventions. Additionally, we will identify potential research gaps and areas that require further investigation, ultimately offering clinicians an updated, evidence-based narrative review.

## DEFINITIONS

Historically, various terms have been used to describe DCI. Therefore, in 2010, Vergouwen et al. proposed more precise definitions. 9 Later, in 2019, a multidisciplinary, international panel established additional terminologies associated with it ( Table 1 ). 10

**Table 1:** Terminologies associated with DCI and aSAH 4 9

Terminology	Definition
Clinical deterioration due to DCI	The occurrence of focal neurological impairment (such as hemiparesis, aphasia, apraxia, hemianopsia, or neglect), or a decrease of at least 2 points on the Glasgow coma scale, either on the total score or on one of its individual components (eye, motor, verbal). Should last for at least 1 hour, is not apparent immediately after aneurysm occlusion, and cannot be attributed to other causes by means of clinical assessment, brain CT or MRI, and appropriate laboratory studies.
Cerebral infarction due to DCI	The presence of cerebral infarction on a CT or MRI scan of the brain within 6 weeks after aSAH, on the latest CT or MRI scan made before death within 6 weeks, or proven at autopsy. Should not be present on the CT or MRI scan between 24 and 48 hours after early aneurysm occlusion and should not be attributable to other causes such as surgical clipping or endovascular treatment. Hypodensities on CT imaging resulting from a ventricular catheter or intraparenchymal hematoma should not be regarded as cerebral infarctions from DCI.
Angiographic cerebral vasospasm	The arterial narrowing of large cerebral vessels is observed on radiological tests such as CT angiography, MRA, or digital subtraction angiography.
Symptomatic cerebral vasospasm	Patients with aSAH develop clinical symptoms attributable to ischemia from visible vasospasm on angiography.

Abbreviations: aSAH, aneurismatic subarachnoid hemorrhage; CT, computed tomography; DCI, delayed cerebral ischemia; MRI, magnetic resonance image; MRA, magnetic resonance angiography.

## PATHOPHYSIOLOGY

Intracranial vasospasm was once considered the sole cause of DCI. However, it involves various underlying pathophysiological mechanisms such as cerebral macro- and microvascular dysfunction, microthrombosis, cortical spreading depolarization (CSD), and neuroinflammation ( Figure 1 ). 11 12 13

**Abbreviations:**
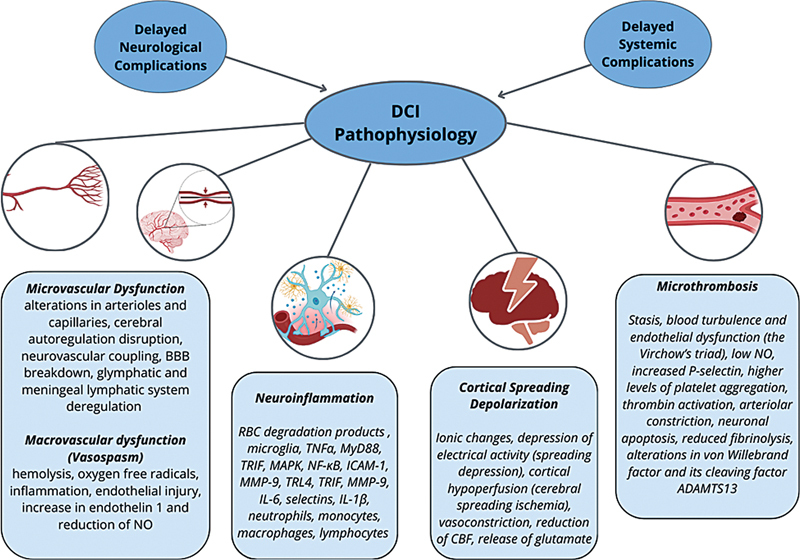
BBB, brain-blood barrier; NO, nitric oxid; ADAMTS13, a disintegrin and metalloproteinase with a thrombospondin type 1 motif, member 13; CBF, cerebral blood flow; RBC, red blood cells; TNF, tumor necrosis factor type α; MyD88, Myeloid differentiation primary response 88; MAPK, mitogen-activated protein kinase; NFKB, nuclear factor kappa B; ICAM1, Intercellular Adhesion Molecule 1; MMP9, Matrix metalloproteinase 9; TRL4, toll-like receptor 4; IL6, interleukin 6; IL 1 β, interleukin 1 β. **Figure 1** Pathophysiology of DCI.

### Vascular dysfunction

**Figure 2:**
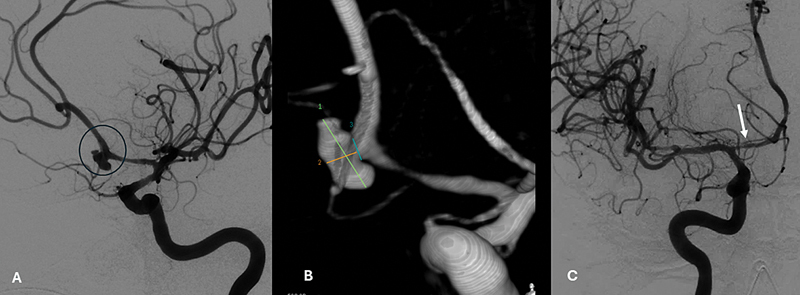
Cerebral vasospasm on digital subtraction angiography (DSA). ( **A** ) DSA showing aneurism in the left anterior cerebral artery (ACA) segment A1. ( **B** ) ACA aneurism after 3D reconstruction. ( **C** ) ACA focal narrowing suggestive of cerebral vasospasm in the same patient.

In macrovascular dysfunction cases, cerebral vasospasm is defined as “the arterial narrowing of large cerebral vessels observed on radiological tests such as CT angiography (CTA), magnetic resonance angiography (MRA), or digital subtraction angiography (DSA)” ( Table 1 ). 9 The release of hemoglobin and hemolysis-mediated degradation products in subarachnoid space triggers reactions with oxygen free radicals, inflammation, endothelial injury, an increase in endothelin-1 (a vasoconstrictor), and a decrease in the vasodilator nitric oxide (NO), 12 resulting in cerebral vasospasm. This phenomenon is detectable only by specialized diagnostic methods ( Figure 2 ) and occurs in up to 70% of patients after aSAH. It typically begins 3 to 4 days after aneurysm rupture, peaks at 7 to 10 days, and resolves within 14 to 21 days. 14 Conversely, DCI is observed in around 30% of aSAH patients and does not always correspond to areas of arterial narrowing. 14 In patients with aneurysmal aSAH who underwent DSA and serial CT scanning, DCI occurred in 3, 10, and 46% of those with no/mild, moderate, or severe angiographic vasospasm, respectively. 11

Furthermore, in microvascular dysfunctions, arterioles and capillaries undergo impaired cerebral autoregulation, neurovascular uncoupling, and breakdown of the blood-brain barrier, which significantly trigger microvascular spasm. 11 Alterations in the meningeal lymphatic and central nervous systems' perivascular glymphatics contribute to cerebral ischemia. The meningeal lymphatic system is responsible for clearing extravasated erythrocytes, so its disruption allows blood products to quickly penetrate brain parenchyma. 14

### Microthrombosis

Aneurysmal subarachnoid hemorrhage affects multiple stages of the blood coagulation cascade, including stasis, blood turbulence, and endothelial dysfunction (the Virchow's triad), low NO, increased P-selectin, higher levels of platelet activation, arteriolar constriction, neuronal apoptosis, reduced fibrinolysis, and alterations in von Willebrand factor, among others. Evidence of microthrombosis has been consistently linked to cerebral ischemia after aSAH in animal models and clinical studies. 11

### Cortical spreading depolarizations

Cortical spreading depolarization is a slowly propagating wave of depolarization that moves outward from its point of origin at a speed of 2 to 5 mm per minute, leading to both a depression of electrical activity (spreading depression) and cortical hypoperfusion (cortical spreading ischemia). 11

Initially, CSD leads to an increase in CBF, resulting in hyperemia, which is followed by a transient phase of oligemia and reduced cerebral perfusion. In pathological conditions, CSD induces arteriolar vasoconstriction and an inversion of neurovascular coupling, generating prolonged and severe hypoperfusion. Moreover, the release of glutamate during CSD can cause neurotoxicity through excessive stimulation and cell death. 11

### Neuroinflammation

The blood in the subarachnoid space triggers an immediate inflammatory response. The release of red blood cell degradation products and high mobility group box-1 (HMGB1) proteins both activate the innate immune cells (particularly microglia), contributing to DCI. 11 Recent reviews highlighted the many factors involved, summarized in Figure 1 . 11 12

It should be noted that all these factors work together, even though they are described separately. For instance, neuroinflammation promotes vasospasm and the occurrence of microthrombosis, both of which contribute to microvascular dysfunction. The pathophysiology of DCI is complex, as it is further influenced by systemic and neurological complications, such as epileptic seizures and hydrocephalus. 13

## PREDICTORS OF DCI

Estimating the risk of DCI is crucial for tailoring the vigilance required for each patient and reducing costs associated with extended hospital stays. The historical predictors of DCI are the volume and extension of bleeding on initial CT, evaluated by the original and modified Fisher (mFisher) scales, 15 and the patient's neurological status upon admission, assessed through the World Federation of Neurological Surgeons (WFNS) and Hunt-Hess scales. 1 4

The first radiological scale was introduced by Fisher et al. in 1980, in which patients classified as grade 3 had a higher risk of vasospasm/DCI compared with those with grade 4. Later, mFisher showed that thick blood and intraventricular hemorrhage (blood in both lateral ventricles) were the strongest predictors of vasospasm and DCI (grade 4). 4

The VASOGRADE scale combines the WFNS and mFisher scores to classify patients with aSAH into green, yellow, and red. Compared with green, the yellow score has an OR of 1.31, and red has an OR of 3.19 for developing DCI/vasospasm. 16 The VASOGRADE scale was also associated with functional outcomes of aSAH patients with high specificity. This could serve as an early indicator for transitioning patients from the intensive care unit (ICU) to the ward, helping to reduce costs and potential medical complications associated with extended internations. 17

Other described predictors of DCI include female gender, diabetes mellitus, early rise in serum C-reactive protein, preexisting hypertension, a high white blood cell count on admission, intracranial infection, smoking, hyperglycemia, hydrocephalus, poor collateral status on DSA, and early systemic inflammatory response syndrome. 18 19 20 Notably, the aneurysm's location and size are not associated with this condition. 18

High levels of cerebrospinal fluid (CSF) lactate and glucose in the first 3 days following aSAH were independent predictors. 21 Dynamic changes in systemic inflammation response index have also been associated with DCI, 22 as have higher levels of admission N-terminal probrain natriuretic peptide (NT-pro BNP), which were also associated with neurogenic cardiac injury. 23

An automated electroencephalography (EEG)-based approach for DCI prediction showed improved accuracy when combined features were analyzed (α-delta ratio and percent α variability, Shannon entropy, and epileptiform discharge burden). 24 Furthermore, transcranial Doppler (TCD) measurements of peak flow velocity in the middle cerebral artery (MCA) above 200 cm/s, combined with EEG epileptiform abnormalities, can predict it better than EEG alone on day 3. 25 In the “International Subarachnoid Aneurysm Trial” (ISAT), DCI incidence was higher after surgical clipping of the aneurysm than after endovascular coiling, probably related to blood vessel manipulation during neurosurgical procedures. 26

The early clot clean rate was associated with reduced incidence of DCI and outcomes at 30 days. 27 Poor arterial collateral status on cranial CT at admission also increased the risk for this condition in a retrospective study. 28 Regardless of absolute values, greater hemoglobin decrement from admission to discharge was independently associated with a higher incidence of DCI and poor functional outcomes. 29

A systematic review identified seven potential biomarkers of clinical DCI: haptoglobin polymorphisms 2–1 and 2–2, ADAM metallopeptidase with thrombospondin type 1 motif 13 (ADAMTS13), neutrophil/lymphocyte ratio, P-selectin, von Willebrand Factor (vWF), and F2-isoprostane in urine. Further studies are needed to standardize cutoff values and to explore if those biomarkers could be used as preventive or therapeutic targets. 30

Neurofilament proteins (NFs) are consistently elevated in serum and CSF upon admission in aSAH patients and may have a relationship with disease severity, prognosis, and mortality. The C-reactive protein (CRP), lactate, microRNAs, estrogen, galectins, D-dimer, neuroglobin, high-mobility group box1 protein (HMGB1), periostin, and glial fibrillary acidic protein (GFAP) also have a potential role regarding DCI and poor prognosis. 31

### Big data and artificial intelligence

The adoption of artificial intelligence and big data analytics may improve the prediction of DCI, which can enhance patient management and treatment strategies. Various machine learning (ML) models, such as the Random Forest, XGBoost, Support Vector Machines, Multilayer Perceptron, Gradient Boosting Decision Trees, and Decision Trees, have been developed and validated across multiple centers. 32 They demonstrate higher accuracy, outperforming standard models and logistic regression scoring systems like the VASOGRADE and the Subarachnoid Hemorrhage International Trialists (SAHIT) prediction models. These models leverage large datasets from clinical records to identify complex patterns and risk factors that may not be apparent through conventional analysis. Moreover, ML models can easily combine clinical variables with image features or biomarkers (such as heart rate variability, matricellular proteins), which increases reliability.

Clinical variables such as age, mFisher, Hunt and Hess score, and external ventricular drain (EVD) placement are significant predictors in ML models. 32 Multicenter collaborations and standardized data sharing are essential for further refining these predictive models, ensuring they can be generalized across diverse patient populations and clinical settings. As these technologies advance, they hold the potential to significantly improve patient outcomes by enabling timely and accurate diagnosis and intervention for DCI following aSAH. 33

## PREVENTION

### Pharmacological therapy

The only pharmacological therapy with confirmed effectiveness and safety in DCI prevention is nimodipine, a dihydropyridine L-type calcium channel antagonist. 5 6 This drug's exact mechanisms of action have been investigated and other effects were proposed beyond vasodilation, including reduction of CSD, increased endogenous fibrinolysis, and reduced microthrombosis. 34 Nimodipine reduces DCI even without improvement of vasospasm, 34 and a meta-analysis of randomized control trials (RCTs), including a total of 1,202 patients, found that nimodipine improved all eight outcome measures: good clinical outcome, mortality, morbidity, death attributed to vasospasm, DCI, cerebral infarction, and rebleeding. 35

Current guidelines recommend starting nimodipine within the first 96 hours of subarachnoid bleeding at a dose of 60 mg, every 4 hours, for 21 days (class I, level of evidence A). 5 If arterial hypotension occurs, the dose may be reduced to 30 mg every 2 to 4 hours. 36 Regarding the route of administration, the randomized “Nimodipine Microparticles to Enhance Recovery While Reducing Toxicity After Subarachnoid Hemorrhage” (NEWTON) trial found no improvements in vasospasm when nimodipine sustained-release microparticles directly into the ventricles were compared with standard oral administration. 37

Recently, it was demonstrated that localized nicardipine release implants (NPRIs), placed around the basal cerebral vasculature, are effective in preventing vasospasm and DCI. 38 A recent RCT including 41 patients showed a lower incidence of cerebral vasospasm, reduced clinical need for rescue therapy, lower rates of new cerebral infarcts, and a higher proportion of favorable functional outcomes in the NPRIs group. 39

One study analyzed the impact of carotid siphon calcification (CSC) on vasospasm and outcomes, identifying a negative impact only in patients not using aspirin. This suggests aspirin may mitigate microcirculatory impairment, indicating potential benefits of aspirin in DCI prevention in patients with aSAH and concomitant carotid atherosclerosis. 40

The administration of clazosentan, magnesium, statins, aspirin, enoxaparin, erythropoietin, fludrocortisone, methylprednisolone, and prophylactic balloon angioplasty have been tested in DCI prevention with negative results. 36 Guidelines from different countries recommend other drugs. For example, tirilazad, a nonglucocorticoid 21-aminosteroid that inhibits lipid peroxidation and free radical production, is approved for treating aSAH in 21 countries. Fasudil has been used in China and Japan since 1995. Furthermore, cilostazol and clazosentan are recommended in Japan to reduce DCI. 34

Ongoing studies are evaluating anakinra, cilostazol, clazosentan, deferoxamine, dexamethasone, IV heparin, isoflurane sedation, ketamine sedation, magnesium-rich artificial CSF, IV milrinone, nadroparine, NPRIs, and intraventricular fibrinolysis with tissue plasminogen activator.

Noteworthy, one systematic review found that pharmaceutical treatments decreased the incidence of both cerebral infarction (relative risk [RR]: 0.83; 95%CI: 0.74–0.93) and poor functional outcome (RR: 0.92; 95%CI: 0.86–0.98). 6 This indicates that the clinical features of DCI may evolve. Clinical diagnosis shows a lower rate of interobserver agreement. In contrast, cerebral infarction observed on neuroimaging is a result of DCI that may not be present in all patients but is strongly correlated with functional outcomes 3 months after aSAH. It also exhibits a high interobserver agreement rate, facilitating diagnosis in sedated and comatose patients. Currently, there is a consensus that cerebral infarction is a preferable primary outcome for observational prevention trials, with the clinical definition of DCI reserved as a secondary outcome. 41

### Euvolemia

Maintaining euvolemia and avoidance of hypervolemia have proven benefits in preventing DCI and improving functional outcomes. It is recommended in current guidelines as patients with documented volume depletion have a higher chance of developing DCI. 2 5 Early goal-directed fluid therapy (EGDT) guided by transpulmonary thermodilution may be an interesting option and was shown to reduce DCI.

Regarding clinical outcomes, in 2014, Mutoh et al. 42 conducted a prospective trial with 160 patients randomized to EGDT guided by preload and cardiac output measurements or conventional therapy guided by central venous pressure and fluid balance. Among those with poor-grade hemorrhages, DCI and the ICU length of stay were significantly reduced with EGDT. Good outcomes (mRs 0–3 in 3 months) were significantly higher in the EGDT group (52 versus 36%; *p* = 0.026). 42

Another prospective, randomized trial with 108 patients achieved similar results: reduced DCI and disability with EGDT. 43 However, liberal fluid administration without goal-directed targets and consequent hypervolemia is not recommended due to the increased risk of pulmonary edema and cardiac complications. 2 5

### Cerebrospinal fluid diversion

Regarding common neurological complications, CSF diversion is used to treat intracranial hypertension and hydrocephalus 44 While external ventricular drains (EVDs) are an important measure to reduce intracranial hypertension after aSAH, they have questionable benefits. Theoretically, the removal of blood clots may reduce DCI. However, aggressive drainage has not been proven to benefit. 45

Lumbar drains have consistently been shown to reduce DCI and improve functional outcomes after aSAH. It also reduces oxidative stress and looks more successful than EVDs for blood clot removal. 46 An ongoing trial in the United States (NCT03065231), now recruiting, aims to answer whether these drains are superior to EVDs for DCI prevention.

More recently, CSF filtration with neurapheresis has shown the potential to reduce clot burden and potentially DCI after aSAH. The fluid is removed, cleaned, and returned to the lumbar spine. However, further studies with larger populations are needed for clarification.

## DIAGNOSIS AND TRIGGERS FOR INTERVENTION

Serial neurological examination is the gold standard for diagnosing DCI in awake patients, which must performed every 2 to 4 hours. 47 For high-grade aSAH patients who are in a coma or sedated, additional methods to detect DCI are recommended, including TCD, DSA, CTA, CT with perfusion (CTP), continuous electroencephalography (cEEG), partial brain tissue oxygenation monitoring (PbTiO2), and cerebral microdialysis (CMD). 48 In 20% of patients diagnosed with DCI-related infarctions, no clinical deterioration was observed before the neuroimaging findings. 36

### Digital subtraction angiography and computed tomography angiography

The use of DSA is the gold standard for detecting vasospasm; however, its availability is limited, particularly in low- and middle-income countries. Vasospasm on DSA can be classified as mild to moderate (grade I: 0–25%; and II: 26–50% narrowing) and severe (grade III [50–75%] and grade IV [> 75%]). 47 48 In contrast, CTA is a less invasive method and has frequently been used instead of DSA. In a meta-analysis of 7 studies, comprising 1,646 arterial segments, CTA had a pooled sensitivity of 82% (95%CI: 68–91%) and a specificity of 97% (95%CI: 93–98%), compared with the gold standard DSA. 49 The disadvantages of CTA include lower accuracy for medium and small vessels, susceptibility to artifacts such as metal clips and coils, and overestimation of arterial narrowing. 47

### Transcranial Doppler ultrasonography

**Figure 3:**
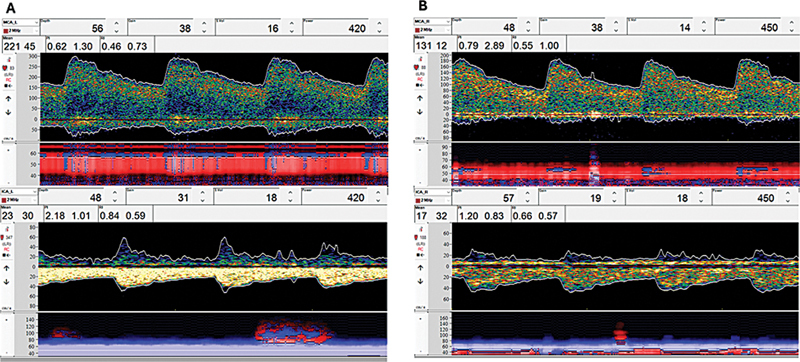
Cerebral vasospasm on transcranial Doppler. ( **A** ) Transcranial Doppler showing severe vasospasm on left middle cerebral artery (MCA) in a patient with aneurismal subarachnoid hemorrhage at 7-days postbleeding: mean flow velocity (MVF) on MCA of 221 cm/s, MFV on left internal carotid artery (ICA) of 30 cm/s, Lindegaard ratio (LR) of 7.3. ( **B** ) Moderate vasospasm on right MCA, with MFV of 131 cm/s, and right ICA MFV of 32 cm/s, LR of 4.09.

As a non-invasive, safe, and quick method, TCD allows real-time bedside assessment of CBF of the intracranial vessels. It is recommended in the period of highest risk for vasospasm and DCI (3–14 days), but this time may be extended in higher risk patients. 5 Furthermore, TCD is most reliable for detecting MCA vasospasms, with less accuracy for other arteries ( Table 2 3 ). 50 A mean flow velocity (MFV) in the MCA > 120 cm/s or an increase ≥ 50 cm/s over 24 hours indicates vasospasm, while an MFV greater than 200 cm/s suggests severe vasospasm ( Figure 3 ).

**Table 2:** Transcranial Doppler criteria for middle cerebral artery vasospasm

Mean flow velocity (cm/s)	Lindegaard index	Interpretation
> 120	≤ 3	Hyperemia
≥ 120	3–4	Light spasm + hyperemia
≥ 120	4–5	Moderate spasm + hyperemia
≥ 120	5–6	Moderate spasm
≥ 180	> 6	Moderate to severe spasm
≥ 200	≥ 6	Severe spasm
> 200	4–6	Moderate spasm + hyperemia
> 200	3–4	Hyperemia + light spasm (often residual)
> 200	< 3	Hyperemia

**Table 3:** Transcranial Doppler criteria for other cerebral arteries (except MCA) vasospasm 50

Artery	Spasms (MFV, cm/s)		
	Possible	Probable	Definitive
ICA	> 80	> 110	> 130
ACA	> 90	> 110	> 120
PCA	> 60	> 80	> 90
BA	> 70	> 90	> 100
VA	> 60	> 80	> 90

Abbreviations: ACA, anterior cerebral artery; BA, basilar artery; ICA, internal carotid artery; MCA, middle cerebral artery; MFV, mean flow velocity; PCA, posterior cerebral artery; VA, vertebral artery.

To prevent misinterpretation due to conditions like anemia and fever, which can cause a hyperdynamic state, evaluation with the Lindegaard ratio (LR) is recommended, calculated by dividing the MFV of the MCA by the MFV of the extracranial ipsilateral internal carotid artery. An LR greater than 3 indicates vasospasm, and greater than 6 suggests severe vasospasm. 51 Several publications have shown high specificity of TCD (94–100%), though it has variable sensitivity (39–96%) compared with DSA in the MCA. 51

Additionally, the absence of a transtemporal window in approximately 20% of individuals may limit the effectiveness of the method in some patients. 52 As such, TCD should ideally be used in conjunction with neurological examinations and other monitoring modalities to improve the accuracy of detecting DCI.

Limitations of the IL include ICA plaques, stenosis, and occlusions, which may influence the blood flow velocities. 53 Furthermore, monitoring blood flow in the ICA in the ICU setting may be hampered by the presence of invasive devices and cervical edema. These factors motivated the study of cerebral vein ultrasound as an alternative for detecting vasospasm.

Mursch et al. 53 prospectively investigated the velocities in the Rosenthal basal vein (RBV), MCA, and extracranial ICA in 66 patients after spontaneous aSAH, and concluded that in patients with increased MCA MFV (above 120 cm/s), those who also had increased velocities in the RBV had a better prognosis. This finding suggests that an increase in the RBV velocities concomitant with an increase in the MCA reflects a state of hyperemia. In turn, patients with normal RBV and increased MCA velocities probably have vasospasm.

Another study prospectively evaluated MCA MFV, MCA peak systolic velocity (PSV), IL using MFV and PSV, and an original arteriovenous index (AVI) between the MCA and the RBV, using MFV and PSV. 54 They compared both IL and AVI with the gold standard DSA in the diagnosis of vasospasm. Interestingly, the AVI showed higher accuracy in the diagnosis of arterial vasospasm compared with the IL. An AVI > 10 (considering MFV) and an AVI > 12 (considering PSV) provided the highest accuracies of 87 and 86%, respectively. Regarding the IL, the accuracy was higher using a threshold of > 3 for MFV (84%) and for PSV (80%). 54

### Computed tomography perfusion

**Figure 4:**
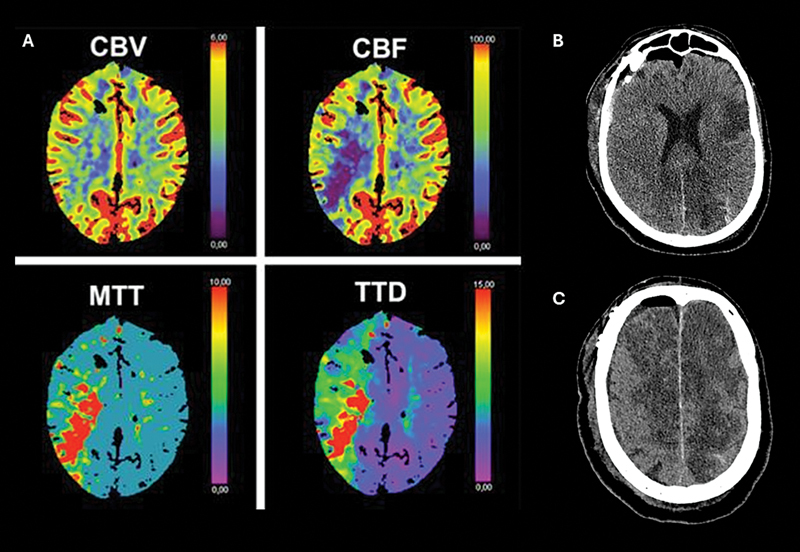
Delayed cerebral ischemia on computed tomography with perfusion images. ( **A** ) Computed tomography with perfusion (CTP) showing prolonged mean transit time (MTT), time to detection (TTD) and reduced cerebral blood flow (CBF) in right parietal lobe, with preserved cerebral blood volume (CBV) in a patient with DCI after aSAH. ( **B** ) Brain computed tomography showing definitive cerebral infarction due to DCI at left frontal and parietal lobes in another patient with aSAH (anterior cerebral artery aneurysm). ( **C** ) Cerebral infarction associated with DCI at anterior cerebral arteries territories bilaterally (anterior cerebral artery aneurysm).

As a relatively fast-performing, minimally invasive, and reasonably cost-effective method, CTP is used to evaluate both macro and microvascularization, as well as cerebral tissue perfusion ( Figure 4 ). Therefore, it plays an important role in predicting and diagnosing DCI, especially for patients with poor grade aSAH who are sedated or comatose, where it is difficult to rely only on serial clinical examinations. Several studies have demonstrated that an increase in the mean transit time (MTT) above 6.5 seconds, and reduction of CBF below 25 mL/100 g/minute, in the appropriate context, has high accuracy and negative predictive value. 47 55 A systematic review of 882 patients confirmed these results. 56

### Continuous electroencephalography

The use of cEEG provides noninvasive, continuous, real-time data on cortical activity, with well-established sensitivity to ischemia. Claassen et al. first demonstrated that a reduction in the α/delta ratio of more than 10% from baseline, and decreased α variability, were the parameters best correlated with early, reversible stages of DCI. 57

In a retrospective study, cEEG monitoring detected DCI and cerebral vasospasm with specificities of 82.9% (95%CI: 66.4–93.4%) and 94.4% (95%CI: 72.7–99.9%), respectively. 58 Additionally, enhanced delta patterns, epileptiform activity, and nonconvulsive status epilepticus are all associated with poor outcomes. 59

To improve the exam's quality, researchers have utilized relative rather than absolute parameters, exclusively artifact-free monitoring, as well as longer monitoring times.

### Near-infrared spectroscopy

Near-infrared spectroscopy (NIRS) is another continuous, noninvasive examination technique that provides information about CBF by estimating intracerebral oxygen saturation. A decrease in regional cerebral oxygen saturation is correlated with ischemia, being a promising tool for DCI detection. However, more studies are needed to confirm the adequate thresholds for diagnosis and triggers for intervention. 60

### Invasive monitoring

Intracranial hypertension is a complication of aSAH associated with alterations in cerebral metabolism leading to ischemia; thus, it requires aggressive treatment. In several neurocritical care patients, maintaining cerebral perfusion pressure (CPP) above 70 mmHg, using invasive intracranial pressure (ICP) and arterial pressure catheters, has been associated with a reduced risk of tissue hypoxia and brain injury. 36

Furthermore, CMD offers insight into the composition of cerebral interstitial fluid and metabolism. A lactate/pyruvate ratio (LPR) > 40 or glucose < 0.5 mM indicates an energy crisis and subsequent hypoxemia/ischemia. 61 Therefore, LPR is considered a warning sign for increased surveillance. This metabolic deterioration can precede cerebral infarction by several hours and is specific for DCI, with lactate > 4 mmol being highly specific. 36 61

The PbTiO2 values can be interpreted as a marker for regional cerebral blood flow, reflecting the balance between oxygen supply, diffusion, and consumption. Cerebral hypoxia (PtiO2 < 20 mm Hg) indicates regional ischemia in SAH patients and suggests intervention together with other parameters. 47 59

Recent guidelines emphasize the lack of evidence to determine ideal triggers for intervention. 5 6 It is important to highlight that TCD, CTA, and DSA identify vasospasm but not DCI. Therefore, ideally, multimodal monitoring methods should be integrated with clinical practice to enhance diagnosis and management ( Table 4 ).

**Table 4:** Diagnosis modalities for delayed cerebral ischemia and cerebral vasospasm after poor-grade SAH 47

Complementary Exam	Pathological alteration
Transcranial Doppler ultrasound	MCA MFV > 120cm/s, LR > 3: vasospasm MCA MFV > 200cm/s or LR > 6: severe vasospasm
Digital subtraction angiography	> 70% narrowing: severe vasospasm
CTA	Arterial narrowing
CTP imaging	MTT > 6.5 seconds; reduction of CBF < 25mL/100 g/minute; or 1.5-fold prolongation compared with baseline indicative for DCI
Continuous electroencephalography	Alpha/delta ration < 50%, reduction in α variability, epileptiform discharges, no reactivity
PbtiO2	< 20 mmHg
Cerebral microdialysis	LPR > 40 glucose < 0.5 mM
Cerebral blood flow monitor	< 20 mL/100 g/minute

Abbreviations: CBF, cerebral blood flow; CTA, computed tomography angiography; CTP, computed tomography with perfusion; DCI, delayed cerebral ischemia; LPR, lactate pyruvate ratio; MCA, middle cerebral artery; MFV, mean flow velocity; MTT, mean transit time; PbTiO2, partial brain tissue oxygenation; SAH, subarachnoid hemorrhage.

## MANAGEMENT OF DCI

Once the diagnosis of DCI is established, prompt interventions are warranted to prevent cerebral infarction and permanent neurological deficits. Guidelines and observational studies from high-income countries show that treating these patients in high-volume centers, with at least 35 aSAH cases per year—ideally 60 or more—leads to improved outcomes. 5 Initially, volume expansion with crystalloids (bolus of 15 mL/kg of isotonic saline) should be administered targeting euvolemia, with potential increase in cardiac output, cerebral perfusion, and cerebral oxygenation analyzed by PbtiO2. 62 63 The so-called “Triple H Therapy”, composed of hypervolemia, arterial hypertension, and hemodilution, is contraindicated. 63

### Hemoglobin optimization

Current guidelines recommend a hemoglobin (Hb) goal above 7 g/dL for patients with aSAH. 6 However, the appropriate threshold for patients with active DCI is unclear. Transfusion of red blood cells increased the partial pressure of PbTiO2 in low-grade aSAH patients with a baseline Hb of 8 g/dL. 64 Conversely, nonguided blood transfusions can lead to complications, poor outcomes, and increased mortality in aSAH patients.

A recent meta-analysis indicates that even mild anemia correlates with cerebral infarction and poor outcomes, particularly with Hb levels between 9 and 10 g/dL. 65 The “Transfusion Strategies in Acute Brain Injured Patients (TRAIN)” trial compared liberal versus restrictive transfusion approaches (maintaining Hb > 9 or > 7 g/dL, respectively) in SAH and traumatic brain injury patients, showing better neurological outcomes in the liberal group. 66 On the other hand, the “Aneurysmal Subarachnoid Hemorrhage – A Red Blood Cell Transfusion and Outcome (SAHaRA)” study showed that a liberal transfusion strategy (mandatory at Hb ≤ 10 g/dL) did not result in a lower risk of an unfavorable neurologic outcome at 12 months compared with a restrictive strategy (optional at Hb ≤ 8 g/dL). 67

### Hemodynamic augmentation

The rationale of hemodynamic augmentation using induced hypertension (IH) or inotropic drugs is to improve CBF and CPP, targeting improvement of cerebral perfusion.

### Induced hypertension

Regarding IH, norepinephrine is widely preferred due to its ability to stimulate α and β receptors, resulting in lower rates of tachycardia and a more predictable hemodynamic response, comparable to phenylephrine. 68 69 Vasopressin is reserved for refractory cases requiring multiple vasoactive drugs. Also, IH is safe in unruptured aneurysms. 70

Nonetheless, there is still a lack of robust evidence regarding IH in preventing and treating DCI. In an observational retrospective trial, Haegens et al. 71 demonstrated the effectiveness of IH in preventing DCI-related cerebral infarction in patients with clinical DCI. The authors 71 included479 patients with clinical signs, and 20% of patients treated with IH developed a DCI-related cerebral infarct, compared with 33% in the no-IH cohort, with statistical significance. Also, IH prevented poor outcome. The RCT “Hypertension Induction in the Management of Aneurysmal Subarachnoid Haemorrhage with Secondary Ischemia (HIMALAIA)” compared the efficacy and safety between IH and no-IH in patients with clinical symptoms of DCI, with a primary outcome of 90-day mRS. However, it was prematurely stopped due to slow recruitment (only 41 patients included) and concerns about the treatment's adverse events. 72

A suggested approach is starting norepinephrine in those with DCI who do not have a basal elevated SBP and/or contraindications (such as a recent myocardial infarction, decompensated congestive heart failure, and pulmonary edema), with an initial target of 160 to 180 mmHg. Neurological reevaluations should be done in 30 minutes intervals and, in cases without improvement, SBP should be augmented up to 220 mmHg, or until there are collateral effects. If there is improvement, that target should be maintained for 48 hours and slowly tapered, based on neurological exams or ancillary tests. If there is recrudescence of symptoms, therapy should resume ( Figure 5 ). 36

**Abbreviations:**
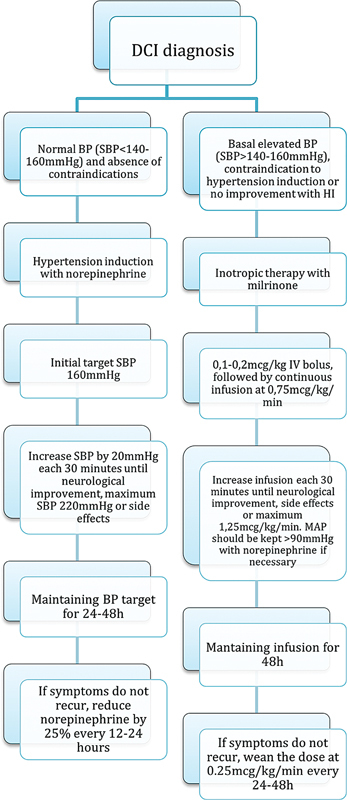
BP, blood pressure; DCI, delayed cerebral ischemia; HI, Hypertension induction; MAP, mean arterial pressure; SBP, systolic blood pressure. **Figure 5** Proposed hemodynamic augmentation strategies for treatment of DCI. 74

### Inotropic therapy

Milrinone, a phosphodiesterase III inhibitor, exhibits positive inotropic and direct vasodilatory effects. It is administered intraarterially, followed by continuous IV infusion. 73 Optimal administration should ideally be paired with hemodynamic monitoring, either minimally invasive or invasive (e.g., transpulmonary thermodilution or pulmonary artery catheter), targeting a cardiac output above 4 L/min/m 2 . 36 In patients with high-grade aSAH, goal-directed hemodynamic is more effective than nontargeted management. 62

In a retrospective case series, Lannes et al. 73 tested the effectiveness of milrinone in reversing DCI and found aSAH-associated vasospasm resolution following the treatment. The study 73 included 88 patients diagnosed from 1999 to 2006, and favorable functional outcomes were achieved in 75% (mRS 0–2). Notably, most participants had low-grade aSAH and needed norepinephrine to maintain SBP and CCP. The Montreal Neurological Hospital's protocol was followed, administering a milrinone bolus of 0.1 to 0.2 mg/kg and continuous infusion of 0.75 to 1.25 mcg/kg/min. When BP decreased below baseline, norepinephrine was used to restore it to previous levels, maintaining mean arterial pressure (MAP) at 90 mmHg. The dosage was progressively increased if symptoms did not improve, with reassessments every 30 minutes ( Figure 5 ). 73

The predictors of refractory vasospasm and DCI despite IV milrinone treatment were investigated retrospectively in a cohort predominantly with high-grade aSAH. Intravenous milrinone was initiated at a dose of 0.25 to 2.5 mcg/kg/min, following diagnosis. 75 Only 21% developed myocardial infarction, and 19% required endovascular rescue therapy (ERT). Moreover, 65% of patients achieved favorable outcomes (mRS 0–2), highlighting milrinone's potential benefits.

The MILRISPASM, a controlled observational study, compared patients with cerebral vasospasm after aSAH who were treated with intravenous milrinone (0.5 µg/kg/min as part of a strict protocol) and IH, to a historical control group that received only IH. Patients treated with milrinone exhibited lower rates of ERT and cerebral infarctions, with better functional outcomes (mRS 0–1). 76 Another retrospective study showed improvement in DCI assessed by TCP after milrinone therapy. 77

A recent publication examined sonographic and clinical outcomes during DCI treatment with either IH or milrinone. The TCD was performed immediately before (t0), and at 45 (t1) and 90 minutes (t2) after therapy initiation, measuring and comparing mean BFV and their kinetics over time. The National Institutes of Health Stroke Scale (NIHSS) and Glasgow coma scale were also evaluated at these prespecified intervals. The analysis of 27 DCI events and 63 spastic vessels demonstrated a significant time-dependent decrease in BFV with these treatments. When comparing therapies, milrinone effectively reduced cerebral BFV, and norepinephrine did not. Clinical improvement was observed with both strategies. This study reinforces milrinone's potential to improve vasospasm, while IH may enhance CBF through collateral vessels. It also confirms that, depending on the therapeutic strategy, clinical and sonographic improvements may not necessarily coincide. 51

### Endovascular rescue therapy

For ERT, both intra-arterial (IA) vasodilating agents and balloon angioplasty (BA) are used. 48 Specific criteria for intervention triggers for ERT are not well established, but they are most used in cases of refractoriness in optimized clinical therapy. In a large observational cohort study, involving over 100,000 patients from an aSAH database, between 2009 and 2018, ERT was linked to lower rates of in-hospital mortality and poor discharge outcomes. 78

### Intra-arterial vasodilators

Cerebral IA vasodilators include fasudil, verapamil, nicardipine, nimodipine, and milrinone. 48 An international online survey with 201 physicians showed that IA nimodipine was the preferred drug. 79 The advantages of IA vasodilators include their diffuse effect and favorable safety profile. Therefore, its pharmacotherapy is recommended for mild to moderate vasospasm (grade I: 0–25% narrowing; and II: 26–50%), particularly in more distal vessels. The disadvantages of IA vasodilators are the shorter duration of effect, potential to increase intracranial pressure due to cerebral vasodilation, and the risk of arterial hypotension, which demands active clinical monitoring during and after the procedure. 48

### Balloon angioplasty

This technique promotes vasodilation through mechanical stretching of the vascular endothelium and disrupting the smooth muscle cells and extracellular matrix of arteries to facilitate increased CBF. 80 It is preferred for treating severe focal vasospasm (grade III: 50–75%; and IV: > 75%) involving proximal intracranial vessels, requiring a minimum vessel diameter of 2 to 2.5 mm. 48

“The Balloon Prophylaxis of Aneurysmal Vasospasm” randomized controlled trial compared prophylactic balloon angioplasty in patients with low-grade aSAH within the first 96 hours and indicated a lower incidence of infarction associated with DCI and neurological deficits in the treated cohort. However, four patients experienced catastrophic vessel rupture, making the study negative and the intervention contraindicated as a preventive strategy. 80 Although it is considered the most effective vasospasm therapy in neurology, caution and experience with balloons are necessary.

In summary, guidelines recommend: 2

- In patients with aSAH and symptomatic vasospasm, elevating systolic BP values may reduce the progression and severity of DCI;
- Use of IA vasodilators and/or cerebral angioplasty may be reasonable in patients with severe vasospasm;
- In patients with aSAH at risk of DCI, prophylactic hemodynamic augmentation should not be performed to reduce iatrogenic patient harm (HOH).

Notably, there is no robust evidence to create recommendations about how to initiate and withdraw IH, and asymptomatic vasospasm in awake patients should not be treated due to potential complications of hemodynamic augmentation. Rather, its detection should lead to increased vigilance and closer neuromonitoring. Milrinone may be useful in refractory cases, and ERT may be employed at any stage of treatment, depending on patients' characteristics and vasospasm location. 36 48

In conclusion, DCI is a critical determinant of long-term outcomes for patients following aSAH. It demands the implementation of close, multimodal neuromonitoring to ensure accurate diagnosis. This condition must be treated as a neurological emergency, requiring the immediate initiation of interventions such as maintaining euvolemia, managing intracranial hypertension, and employing endovascular therapy for refractory cases. We must address several questions and evidence gaps including intervention triggers, blood pressure targets, the efficacy of hemodynamic augmentation, intrathecal vasodilators antiplatelet therapy or low-dose heparin to prevent microthrombosis, oxygen and microdialysis targets to improve brain perfusion, inhalational anesthetics as neuroprotective agents, stellate ganglion block to prevent vasospasm, optimal hemoglobin threshold, CSF drainage efficacy, and the most effective prevention strategies, among others. These efforts can significantly reduce the incidence of DCI and enhance long-term patient outcomes.
